# Nonlinear Relationship Between the Triglyceride–Glucose Index and All‐Cause and Cardiovascular Mortality in Diabetes Mellitus Patients With Hypertension: A National Cohort Study

**DOI:** 10.1155/ije/4467241

**Published:** 2026-05-20

**Authors:** Yuanyuan Zhao, Dongjie Du, Zhi Liu

**Affiliations:** ^1^ Department of Emergency, Xuanwu Hospital, Capital Medical University, Beijing, China, ccmu.edu.cn; ^2^ Department of Cardiology, Beijing Friendship Hospital, Capital Medical University, Beijing, China, ccmu.edu.cn

**Keywords:** diabetes, hypertension, mortality, triglyceride–glucose index

## Abstract

**Background:**

The impact of the triglyceride–glucose (TyG) index on mortality in diabetes patients with hypertension remains unclear.

**Methods:**

This retrospective cohort study included 3296 participants from the National Health and Nutrition Examination Survey (2001–2018). The TyG index was calculated as Ln [fasting triglycerides (mg/dL) × fasting glucose (mg/dL)/2]. Multivariable Cox proportional hazards models adjusted for demographic and clinical confounders were used to estimate mortality risks. Restricted cubic spline analysis assessed nonlinear relationships between TyG index and mortality risk. Stratified analyses and interaction tests were conducted based on gender, age, race, body mass index, smoking status, and the presence of cardiovascular disease.

**Results:**

A total of 3296 individuals were ultimately included in the study cohort. Over a median follow‐up of 82 months (IQR, 44–129), 912 participants (27.7%) died, with 315 (9.6%) from cardiovascular disease. A nonlinear association between TyG index and mortality was observed, with inflection points at 8.9 for all‐cause mortality and 9.0 for cardiovascular mortality. Hazard ratios (95% CI) were 0.85 (0.62–1.16) and 1.38 (1.14–1.67) for all‐cause mortality and 0.70 (0.44–1.01) and 1.78 (1.27–2.48) for cardiovascular mortality, below and above these points, respectively. In participants aged < 65 years, elevated TyG index levels showed significantly greater susceptibility to all‐cause mortality [HR = 1.69 (1.20–2.39), *p* < 0.003, interaction *p* = 0.01].

**Conclusion:**

In this national cohort of adults with diabetes and hypertension, the TyG index showed a nonlinear association with mortality: the risk of all‐cause death increased above TyG = 8.9 and cardiovascular death above TyG = 9.0; associations were stronger among participants under 65 years old.

## 1. Introduction

Diabetes mellitus and hypertension are among the most prevalent cardiometabolic disorders worldwide [1, 2] and frequently coexist. Epidemiological evidence indicates that nearly half of individuals with diabetes also have hypertension, underscoring the substantial overlap between these two conditions [3–6]. Importantly, individuals with concurrent diabetes and hypertension experience substantially higher risks of all‐cause and cardiovascular mortality compared with those with either condition alone [7–10]. This combined disease state reflects persistent metabolic dysregulation and vascular injury, contributing to accelerated atherosclerosis and adverse cardiovascular outcomes [11]. Despite the markedly elevated risk in this population, prognostic indicators that specifically characterize mortality risk in individuals with coexisting diabetes and hypertension remain insufficiently investigated.

The triglyceride–glucose (TyG) index, a simple surrogate marker of insulin resistance (IR), has been associated with incident diabetes, hypertension, and increased all‐cause and cardiovascular mortality in general population‐based studies [12–14]. However, most prior TyG–mortality analyses have been conducted in community‐based or general populations, without focusing specifically on individuals already burdened by established cardiometabolic multimorbidity, such as coexisting diabetes and hypertension. Importantly, the coexistence of diabetes and hypertension represents a distinct and amplified cardiometabolic phenotype rather than a simple additive condition. IR, vascular remodeling, endothelial dysfunction, and neurohormonal activation are often more pronounced in this dual‐disease state, potentially modifying the magnitude and shape of TyG‐related risk associations. Given the markedly elevated cardiovascular risk in this population, whether the TyG index retains independent prognostic value among individuals with coexisting diabetes and hypertension remains unclear. Furthermore, it is unknown whether the association between TyG and mortality in this high‐risk subgroup follows a linear pattern or exhibits potential threshold effects that may differ from those observed in general populations. Clarifying this association may provide clinically meaningful insights for risk stratification in this high‐risk population.

Therefore, we aimed to examine the association between the TyG index and both all‐cause and cardiovascular mortality among adults with coexisting diabetes and hypertension. In addition, we explored potential nonlinear relationships and threshold effects to better characterize risk patterns in this specific population. From a broader translational perspective, improving risk stratification using metabolic biomarkers may also inform future therapeutic strategies, including emerging approaches such as advanced drug delivery systems [15, 16].

## 2. Methods

### 2.1. Study Population and Design

**FIGURE 1:**
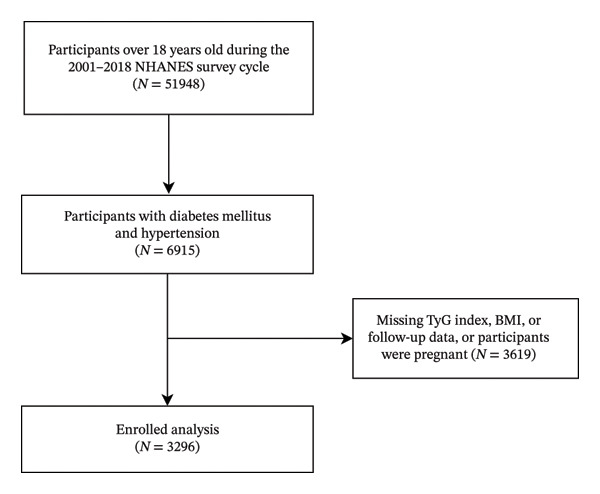
Flowchart of the study.

All data were obtained from the National Health and Nutrition Examination Survey (NHANES), a comprehensive nationwide health survey project that employs a stratified multistage sampling design to obtain representative samples of the U.S. population, with the primary objective of assessing the health and nutritional status of Americans. The survey protocol received approval from the NCHS institutional review board, and all participants provided written informed consent (available from: https://www.cdc.gov/nchs/nhanes/index.htm). The study utilized data from the NHANES database spanning the years 2001–2018. Participants aged ≥ 18 years were eligible for inclusion. Individuals were excluded if they had missing fasting triglyceride or fasting glucose measurements required for calculation of the TyG index, missing BMI data, missing mortality follow‐up information, or were pregnant at baseline. After applying these criteria, 3296 participants were included in the final analysis (Figure 1) [17, 18].

### 2.2. Definition of Diabetes Mellitus and Hypertension

Diabetes mellitus (T2D) was defined as any of the following: glycated hemoglobin (HbA1c) ≥ 6.5%, fasting plasma glucose ≥ 126 mg/dL, self‐reported physician diagnosis of diabetes, or self‐reported use of insulin or glucose‐lowering medications.

Hypertension was defined as systolic blood pressure (SBP) ≥ 130 mmHg or diastolic blood pressure (DBP) ≥ 80 mmHg measured at the baseline examination, self‐reported physician diagnosis of hypertension, or self‐reported use of antihypertensive medications.

Participants meeting both criteria were classified as having coexisting diabetes and hypertension.

### 2.3. Exposure

The TyG index was calculated using the commonly accepted formula: Ln [fasting triglycerides (mg/dL) × fasting glucose (mg/dL)/2], as previously described [19]. Fasting triglycerides and glucose were measured in participants who had fasted for 8–24 h. Participants were grouped into tertiles [Q1(6.80–8.85), Q2(8.85–9.40), Q3(9.40–12.55)] based on TyG index values.

### 2.4. Demographic Characteristics and Covariates

Demographic and health‐related variables, including age, gender, race, educational levels, smoking status, alcohol use, family history of CVD (family CVD) and diabetes mellitus (family DM), diabetes mellitus (DM), presence of hypertension, coronary heart disease (CHD), angina, stroke, heart attack, congestive heart failure (CHF), and use of medications such as antihypertension drugs and lipoprotein‐lowering drugs and glucose‐lowering drugs, were collected through standardized self‐report questionnaires. Education levels were categorized as less than high school (< 12), high school (12), and college or above (> 12). SBP, DBP, weight, and height measurements were acquired using standard methods in the mobile examination center. Smoking status was dichotomized as current smoker or nonsmoker. The history of CVD was ascertained through self‐reported instances of angina pectoris, CHD, heart attack, CHF, and stroke. BMI was calculated as weight (kg) divided by height squared (m^2^). Clinical indicators such as total cholesterol (TC), low‐density lipoprotein cholesterol (LDL‐C), high‐density lipoprotein cholesterol (HDL‐C), triglyceride (TG), uric acid (UA), fasting blood glucose (FBG), hemoglobin A1c (HbA1c), alanine aminotransferase (ALT), total bilirubin (TBiL), and aspartate aminotransferase (AST) were measured in the NHANES laboratory. Estimated glomerular filtration rate (eGFR) was calculated using the chronic kidney disease epidemiology collaboration (CKD‐EPI) equation [20]. Hyperlipidemia was defined as triglyceride ≥  150 mg/dL, HDL‐C  <  40 mg/dL in males (50 mg/dL in females), LDL‐C  ≥  130 mg/dL, TC  ≥  200 mg/dL, or being treated for hyperlipidemia [21].

### 2.5. Ascertainment of Mortality

The research examined all‐cause and cardiovascular mortality as primary outcomes. All‐cause mortality was specifically characterized as fatalities resulting from heart diseases and other causes. Cardiovascular mortality, on the other hand, was defined as deaths attributed to heart diseases and cerebrovascular diseases in accordance with the International Classification of Diseases. The NHANES mortality records were integrated with decedent information from the National Death Index of the National Center for Health Statistics (NCHS) through December 31, 2019. This integration process employed a statistical matching technique to link the datasets. For those seeking more details about mortality‐related variables, the CDC provides a comprehensive resource at https://www.cdc.gov/nchs/data-linkage/mortality.htm.

### 2.6. Statistical Analysis

Continuous variables were presented as mean (standard deviation), and categorical variables were presented as count (percentage). The study population was stratified into TyG index tertiles (Q1–Q3) for analysis. Group differences were assessed using the Wilcoxon‐rank test, chi‐square test, or Kruskal–Wallis test as deemed appropriate. Cox proportional hazards analyses were conducted to examine the relationship between TyG and all‐cause and cardiovascular mortality and calculate hazard ratios (HRs) and 95% confidence intervals (CIs). Univariable and multivariable models were constructed, with age interaction evaluated: the crude model remained unadjusted; Model 1 was adjusted for age, gender, and race; Model 2 was adjusted for age, race, gender, education, LDL‐C, HDL‐C, alcohol use, smoking status, BMI, ALT, AST, TBiL, UA, family DM, family CVD, lipoprotein‐lowering drugs, eGFR, and CVD. Covariates were selected based on prior clinical evidence and the change‐in‐estimate criterion. Specifically, variables previously demonstrated to be associated with all‐cause or cardiovascular mortality were considered potential confounders. In addition, variables that changed the primary exposure effect estimate by more than 10% after inclusion in the model were retained as confounding factors. In addition to reporting HRs, we calculated 10‐year predicted risks of cardiovascular mortality based on the fitted Cox model. Absolute risk differences (ARDs) between TyG ≤ 9.0 and TyG > 9.0 were derived from these model‐based estimates. Restricted cubic spline (RCS) analyses were performed using the “rcssci” package to examine nonlinear associations between the TyG index and mortality outcomes, with four knots placed at the 5th, 35th, 65th, and 95th percentiles of the TyG index distribution. When nonlinearity was detected, we applied segmented Cox regression using a two‐step recursive method to identify inflection points. The inflection point was defined as the value corresponding to maximum likelihood. HRs and 95% CIs were calculated for segments on either side of the threshold. This study conducted stratification analyses and interaction tests for age, gender, race, BMI, CVD, and smoking status to assess the stability of the results. All statistical analyses were conducted using *R* software (Version 4.3.0), with statistical significance defined as *p* < 0.05.

### 2.7. Sensitivity Analyses

Follow‐up time was calculated from baseline to death or censoring in months. In the primary analysis, cause‐specific Cox proportional hazards models were used. For cardiovascular mortality, noncardiovascular deaths were treated as censored observations. As a sensitivity analysis to account for competing risks, Fine–Gray models were applied using the “cmprsk” *R* package, treating noncardiovascular deaths as competing events. Because missing covariate data were present, competing‐risk analyses were conducted using multiple imputation. The proportional hazards assumption was assessed using Schoenfeld residuals. Variables violating the proportional hazards assumption (BMI, ALT, lipoprotein‐lowering drugs, and eGFR) were modeled using time‐dependent Cox regression by including time‐interaction terms. Missing covariate data were handled using complete‐case analysis in the primary models. As a sensitivity analysis, multiple imputation using the “mice” *R* package was performed to account for missing data. Imputed datasets were combined using Rubin’s rules. To assess the robustness of our findings, we excluded participants who died within the first 1 year of follow‐up to reduce the potential for reverse causality. To assess the robustness of the observed associations to potential unmeasured confounding, we calculated E‐values based on the fully adjusted HRs from the two piecewise Cox proportional hazards models. In this study, all E‐values were greater than 2. Detailed results of the time‐dependent Cox models, missing data pattern, multiple imputation analyses, competing‐risk analyses, and analyses excluding deaths within the first year are provided in the Supporting Materials (Tables S1–S8).

## 3. Results

### 3.1. Baseline Characteristics of Study Participants

The baseline characteristics of the cohort study are presented in Table 1. A total of 3296 participants were included, with a mean age of 63.28 years, of which 51.5% were male. The average TyG index was 9.18 (0.75). Compared to participants in the lowest tertile, those in the highest tertile tended to be younger but exhibited higher rates of smoking, Mexican ethnicity, and obesity. Additionally, they had higher levels of blood pressure and a greater proportion of family history of diabetes, history of dyslipidemia, and history of diabetes medication use. Biochemical test results revealed that the high TyG index group showed significantly elevated levels of FPG, HbA1c, eGFR, LDL‐C, TC, TG, and ALT compared to the low TyG index group. Conversely, the low TyG index group demonstrated a higher prevalence of lipid‐lowering and antihypertensive medication use.

**TABLE 1:** Baseline characteristics according to the TyG index tertiles.

Variable	Tertiles of TyG index				*p*
	Total	Q1 (6.80–8.85)	Q2 (8.85–9.40)	Q3 (9.40–12.55)	
Number	3296	1102	1094	1100	
Age (year)	63.28 (12.77)	64.97 (12.78)	64.01 (12.24)	60.87 (12.94)	< 0.001
Gender (male), *n* (%)	1698 (51.5)	573 (52.0)	523 (47.8)	602 (54.7)	0.005
Race, *n* (%)					< 0.001
Mexican American	577 (17.5)	126 (11.4)	191 (17.5)	260 (23.6)	
Non‐Hispanic Black	845 (25.6)	414 (37.6)	236 (21.6)	195 (17.7)	
Non‐Hispanic White	1285 (39.0)	372 (33.8)	464 (42.4)	449 (40.8)	
Other Hispanic	306 (9.3)	85 (7.7)	110 (10.1)	111 (10.1)	
Other race—including multiracial	283 (8.6)	105 (9.5)	93 (8.5)	85 (7.7)	
Education, *n* (%)					0.095
< 12	1170 (35.6)	361 (32.8)	390 (35.7)	419 (38.2)	
12	979 (29.8)	333 (30.3)	322 (29.5)	324 (29.5)	
> 12	1138 (34.6)	405 (36.9)	379 (34.7)	354 (32.3)	
Alcohol use, *n* (%)	2411 (81.9)	814 (82.8)	794 (81.2)	803 (81.6)	0.626
Smoking status, *n* (%)	1659 (50.4)	532 (48.5)	534 (48.8)	593 (54.0)	0.014
BMI, kg/m^2^	32.22 (7.38)	31.41 (7.96)	32.50 (7.20)	32.75 (6.89)	< 0.001
SBP, mmHg	136.19 (20.09)	135.77 (20.18)	136.04 (20.02)	136.77 (20.07)	0.499
DBP, mmHg	70.34 (13.73)	69.10 (13.73)	70.21 (13.51)	71.73 (13.83)	< 0.001
TC, mmol/L	4.86 (1.19)	4.46 (1.02)	4.82 (1.05)	5.29 (1.33)	< 0.001
LDL‐C, mmol/L	2.74 (0.98)	2.55 (0.87)	2.82 (0.95)	2.87 (1.09)	< 0.001
HDL, mmol/L	1.29 (0.40)	1.48 (0.46)	1.28 (0.31)	1.10 (0.30)	< 0.001
TG, mmol/L	1.88 (1.70)	0.93 (0.30)	1.57 (0.42)	3.13 (2.41)	< 0.001
FPG, mg/dL	151.95 (61.13)	118.51 (25.16)	140.82 (37.20)	196.51 (77.20)	< 0.001
TyG, mean (SD)	9.18 (0.75)	8.42 (0.34)	9.13 (0.16)	10.00 (0.55)	< 0.001
HbA1c, %	7.09 (1.70)	6.44 (1.03)	6.80 (1.26)	8.02 (2.15)	< 0.001
eGFR, mL/min/1.73 m^2^	80.47 (25.35)	78.86 (26.10)	79.62 (24.55)	82.92 (25.21)	< 0.001
ALT, IU/L	26.47 (17.26)	23.42 (14.07)	26.20 (17.31)	29.81 (19.40)	< 0.001
AST, IU/L	26.24 (14.56)	25.41 (13.83)	25.87 (13.91)	27.45 (15.79)	0.003
TBiL, IU/L	11.77 (5.06)	12.00 (5.29)	11.58 (4.95)	11.73 (4.91)	0.154
UA, mmol/L	354.22 (93.94)	351.36 (92.64)	354.93 (91.54)	356.38 (97.53)	0.437
Hyperlipidemia, *n* (%)	2901 (88.0)	852 (77.3)	974 (89.0)	1075 (97.7)	< 0.001
Family CVD, *n* (%)	495 (15.0)	146 (13.2)	176 (16.1)	173 (15.7)	0.128
Family DM, *n* (%)	2015 (61.1)	657 (59.6)	633 (57.9)	725 (65.9)	< 0.001
CVD, *n* (%)	905 (27.5)	325 (29.5)	276 (25.2)	304 (27.6)	0.081
Lipoprotein‐lowering drugs, *n* (%)	1648 (50.0)	585 (53.1)	539 (49.3)	524 (47.7)	0.033
Antihypertension drug, *n* (%)	2486 (75.5)	854 (77.6)	836 (76.4)	796 (72.5)	0.015
Glucose‐lowering drug (%)	1918 (58.2)	605 (55.0)	623 (56.9)	690 (62.8)	< 0.001

*Note:* TyG: triglyceride–glucose index, BMI: body mass index, SBP: systolic blood pressure, DBP: diastolic blood pressure, HDL‐C: high‐density lipoprotein cholesterol, LDL‐C: low‐density lipoprotein cholesterol, TC: total cholesterol, TG: triglyceride, UA: uric acid, FBG: fasting blood glucose, HbA1c: hemoglobin A1c, ALT: alanine aminotransferase, TBiL: total bilirubin, AST: aspartate aminotransferase, eGFR: estimated glomerular filtration rate, CVD: cardiovascular disease.

### 3.2. Relationships of TyG Index With Mortality

During a median follow‐up of 82 months (IQR, 44–129), a total of 912 (27.7%) all‐cause deaths and 315 (9.6%) CVD‐related death events were recorded (Table 1). To investigate the independent relationship between TyG index levels and mortality risk, we established three Cox proportional hazards regression models (Table 2). In the most comprehensive Model 2, we adjusted for multiple potential confounding factors, including demographic characteristics (age, race, gender, and education level), lifestyle factors (alcohol use and smoking), clinical indicators (BMI, LDL‐C, HDL‐C, ALT, AST, TBiL, UA, and eGFR), medical history (CVD), family history (CVD and diabetes), and medication use (lipid‐lowering drugs). When TyG was treated as a categorical variable, the adjusted HR (95% CI) for all‐cause mortality was 1.02 (0.85–1.22) and 1.19 (0.98–1.45) for Q2 and Q3 groups, respectively, compared to Q1 (reference) (*p* for trend = 0.07). For cardiovascular mortality, the corresponding HRs were 0.99 (0.73–1.35) and 1.2 (0.86–1.68) (*p* for trend = 0.27). Furthermore, when TyG was treated as a continuous variable, each one‐unit increase in TyG index was associated with an 18% increase in all‐cause mortality risk (HR 1.18, 95% CI 1.03–1.35) and a 23% increase in cardiovascular mortality risk (HR 1.23, 95% CI 0.98–1.56). At 10 years, the predicted risk of cardiovascular mortality was 8.77% among participants with TyG ≤ 9.0 and 10.05% among those with TyG > 9.0, corresponding to an ARD of 1.28% points. These findings indicate that although the relative hazard was elevated above the threshold, the corresponding absolute risk increase was modest.

**TABLE 2:** HRs (95% CIs) for mortality according to the TyG index.

	**TyG (continuous)**		**TyG (categories)**					
			**Q1 (6.80–8.85)**	**Q2 (8.85–9.40)**		**Q3 (9.40–12.55)**		***p* for trend**
**All-cause mortality**	**HR (95% CI)**	***p***		**HR (95% CI)**	***p***	**HR (95% CI)**	***p***	

Crude model	0.96 (0.88, 1.05)	0.34	Ref.	0.87 (0.74, 1.02)	0.09	0.91 (0.78, 1.07)	0.25	0.30
Model 1	1.12 (1.02, 1.24)	0.02	Ref.	0.89 (0.76, 1.05)	0.18	1.14 (0.97, 1.34)	0.12	0.09
Model 2	1.18 (1.03, 1.35)	0.02	Ref.	1.02 (0.85, 1.22)	0.86	1.19 (0.98, 1.45)	0.08	0.07

*CVD mortality*								
Crude model	0.91 (0.79, 1.06)	0.24	Ref.	0.8 (0.61, 1.05)	0.11	0.81 (0.62, 1.06)	0.13	0.14
Model 1	1.13 (0.95, 1.33)	0.16	Ref.	0.86 (0.65, 1.13)	0.27	1.11 (0.84, 1.46)	0.47	0.45
Model 2	1.23 (0.98, 1.56)	0.08	Ref.	0.99 (0.73, 1.35)	0.97	1.2 (0.86, 1.68)	0.28	0.27

*Note:* Crude model: non‐adjusted. Model 1: adjusted for age, gender, and race. Model 2: adjusted for age, race, gender, education, alcohol use, smoking status, BMI, LDL‐C, HDL‐C, ALT, AST, TBiL, UA, family CVD, family DM, lipoprotein‐lowering drugs, eGFR, and CVD.

### 3.3. Nonlinear Relationship Analyses

Given the continuous nature of the TyG index, we conducted a nonlinear relationship analysis. The results (as shown in Figure 2) revealed a nonlinear association between the TyG index and both all‐cause and cardiovascular mortality rates. This finding was obtained after controlling for multiple potential confounding factors, including demographic characteristics, lifestyle, clinical indicators, family history, and medication use. We found that the relationship curves for all‐cause mortality and cardiovascular mortality exhibited distinct inflection points, occurring at TyG index values of 8.9 and 9.0, respectively. HRs were expressed per 1‐unit increase in TyG within each segment. The log‐likelihood ratio test results (*p*‐values of 0.026 and 0.001, respectively) further supported the existence of this nonlinear relationship. The two‐piecewise Cox proportional hazards model showed that to the right of the inflection points, each 1‐unit increase in the TyG index was significantly associated with increased mortality risk: for all‐cause mortality, HR 1.38 (95% CI 1.14–1.67, *p* = 0.001) and for cardiovascular mortality, HR 1.78 (95% CI 1.27 to 2.48, *p* = 0.001). However, to the left of the inflection points, each 1‐unit increase in the TyG index was not significantly associated with mortality risk: all‐cause mortality HR 0.85 (95% CI 0.62–1.16) and cardiovascular mortality HR 0.70 (95% CI 0.44–1.01). Detailed results are presented in Table 3.

FIGURE 2Association between TyG and all‐cause/cardiovascular mortality. Adjusted for age, race, gender, education, alcohol use, smoking status, BMI, LDL‐C, HDL‐C, ALT, AST, TBiL, UA, family CVD, family DM, lipoprotein‐lowering drugs, eGFR, and CVD. (a) All‐cause mortality. (b) Cardiovascular mortality.

**Figure:**
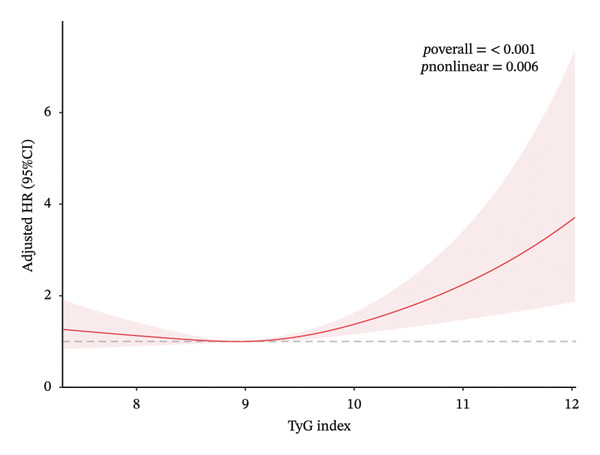
(a)

**Figure:**
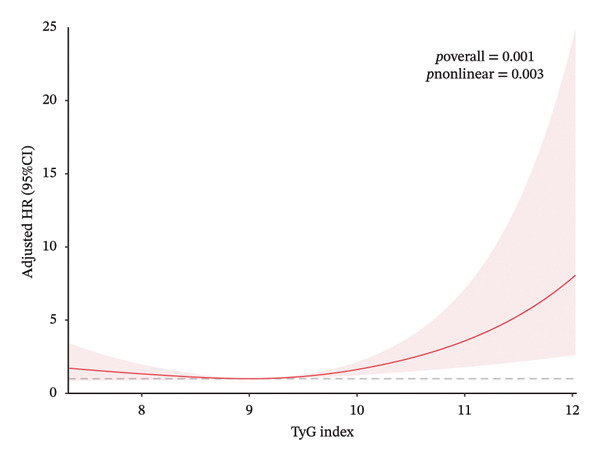
(b)

**TABLE 3:** Threshold effect analysis of TyG index on all‐cause and cardiovascular mortality in diabetes patients with hypertension.

	**HR (95% CI)**	**p**

*All-cause mortality*		
Fitting by the standard Cox proportional risk model	1.18 (1.03, 1.36)	0.015

*Fitting by two-piecewise Cox proportional risk model*		
Inflection point	8.9	
TyG index < 8.9	0.85 (0.62, 1.16)	0.305
TyG index ≥ 8.9	1.38 (1.14, 1.67)	0.001
*p* for log‐likelihood ratio	0.026	

*Cardiovascular mortality*		
Fitting by the standard Cox proportional risk model	1.24 (0.98, 1.57)	0.071

*Fitting by two-piecewise Cox proportional risk model*		
Inflection point	9.0	
TyG index < 9.0	0.70 (0.44, 1.01)	0.120
TyG index ≥ 9.0	1.78 (1.27,2.48)	0.001
*p* for log‐likelihood ratio	0.007	

*Note:* HR was estimated using a two‐piecewise Cox proportional risk model on both sides of the inflection point (all‐cause mortality: 8.9; cardiovascular mortality: 9.0) and adjusted for confounders. Adjusted for age, race, gender, education, alcohol use, smoking status, BMI, LDL‐C, HDL‐C, ALT, AST, TBiL, UA, family CVD, family DM, lipoprotein‐lowering drugs, eGFR, and CVD.

### 3.4. Stratified Analyses

In our study, participants were categorized into high and low TyG index groups based on previously determined inflection points. The cutoff point for all‐cause mortality was 8.9, and for cardiovascular mortality was 9.0. To thoroughly investigate the relationship between the TyG index and mortality, we conducted a series of stratified and interaction analyses, covering factors such as gender, age, race, BMI, CVD, and smoking status. The analysis results showed that in most subgroups, the relationship between the TyG index and mortality remained consistent, with no significant interaction effects observed (*p* for interaction all greater than 0.05). However, age emerged as an exception (*p* for interaction = 0.01) (detailed in Tables 4 and 5), whereas no significant interactions were observed by gender, BMI, CVD, smoking status, or race. The age subgroup analysis revealed an interesting phenomenon: The impact of the TyG index on all‐cause mortality differed across age groups. Specifically, for individuals under 65 years, a higher TyG index was significantly associated with an increased risk of all‐cause mortality (HR 1.69, 95% CI 1.20–2.39, *p* = 0.003), whereas for individuals 65 years and older, no significant association was observed between the TyG index and all‐cause mortality risk (HR 1.06, 95% CI 0.88–1.29, *p* = 0.52).

**TABLE 4:** Stratified analyses of the associations between TyG and all‐cause mortality.

TyG index	HR (95% CI)		*p*	*p* for interaction
	< 8.9	≥ 8.9		
Age				0.01
< 65	Ref.	1.69 (1.20,2.39)	0.003	
≥ 65	Ref.	1.06 (0.88,1.29)	0.52	
BMI				0.92
< 24	Ref.	1.14 (0.74,1.75)	0.55	
≥ 24	Ref.	1.18 (0.98,1.42)	0.08	
CVD				0.34
No	Ref.	1.22 (0.98,1.51)	0.08	
Yes	Ref.	1.15 (0.89,1.49)	0.30	
Gender				0.12
Female	Ref.	1.36 (1.05,1.76)	0.02	
Male	Ref.	1.06 (0.85,1.32)	0.62	
Race				0.06
Mexican American	Ref.	1.17 (0.68,2.00)	0.57	
Non‐Hispanic Black	Ref.	1.39 (1.00,1.93)	0.05	
Non‐Hispanic White	Ref.	1.07 (0.85,1.34)	0.57	
Other Hispanic	Ref.	1.24 (0.46, 3.32)	0.67	
Other race	Ref.	1.64 (0.71,3.80)	0.25	
Smoking status				0.78
No	Ref.	1.2 (0.93,1.54)	0.16	
Yes	Ref.	1.15 (0.92,1.44)	0.21	

**TABLE 5:** Stratified analyses of the associations between TyG and cardiovascular mortality.

TyG index	HR (95% CI)		*p*	*p* for interaction
	< 9.0	≥ 9.0		
Age				0.29
< 65	Ref.	1.26 (0.70,2.30)	0.44	
≥ 65	Ref.	1.06 (0.79,1.43)	0.71	
BMI				0.97
< 24	Ref.	1.05 (0.49, 2.23)	0.90	
≥ 24	Ref.	1.15 (0.86,1.54)	0.35	
CVD				0.97
No	Ref.	1.14 (0.78,1.67)	0.50	
Yes	Ref.	1.19 (0.81,1.76)	0.37	
Gender				0.44
Female	Ref.	1.34 (0.89,2.01)	0.16	
Male	Ref.	1.06 (0.74,1.52)	0.75	
Race				0.1
Mexican American	Ref.	3.14 (0.97,10.11)	0.06	
Non‐Hispanic Black	Ref.	0.99 (0.58,1.68)	0.97	
Non‐Hispanic White	Ref.	0.97 (0.67,1.40)	0.87	
Other Hispanic	Ref.	3.58 (0.54, 23.80)	0.19	
Other race	Ref.	1.41 (0.26, 7.65)	0.69	
Smoking status				0.92
No	Ref.	1.22 (0.82,1.80)	0.32	
Yes	Ref.	1.15 (0.79,1.66)	0.47	

### 3.5. Sensitivity Analyses

Additional details of sensitivity analyses are provided in the Supporting Materials (Tables S1–S8). In sensitivity analyses using Fine–Gray competing‐risk regression with multiple imputation, the association between TyG and cardiovascular mortality remained statistically significant in the fully adjusted model (sHR 1.24, 95% CI 1.01–1.50; *p* = 0.04), consistent with the primary findings (Table S7). Additionally, time‐dependent Cox models accounting for violations of the proportional hazards assumption yielded consistent results (Tables S1 and S2). The distribution of missing data is presented in Table S3, and baseline characteristics after multiple imputation are shown in Table S4. Results from multiple imputation analyses were consistent with the primary complete‐case analyses (Tables S5 and S6). In addition, exclusion of deaths within the first year of follow‐up did not materially change the overall findings, supporting the robustness of the association between TyG and mortality (Table S8).

## 4. Discussion

This study examined the relationship between TyG index and mortality risks in patients with both diabetes and hypertension. To our knowledge, this study provides evidence of a nonlinear association between baseline TyG index and the risks of all‐cause and cardiovascular mortality in this specific patient population. The associations appeared to differ on either side of internally identified inflection points. In addition, the TyG index was associated with increased risk of all‐cause mortality among younger participants.

The exact physiological pathway linking TyG index to mortality is not yet fully understood. The TyG index is widely regarded as a reliable surrogate marker of IR [22]. IR is characterized by reduced cellular responsiveness to insulin, leading to impaired glucose utilization and metabolic dysregulation, and is strongly associated with increased risks of metabolic disorders and cardiovascular disease [23]. Elevated TyG levels reflect underlying IR and have been linked to chronic inflammation and endothelial dysfunction, both of which promote atherosclerotic plaque formation [24, 25]. In addition, elevated triglyceride‐rich lipoproteins (TRLs) and remnant cholesterol contribute independently to atherosclerotic cardiovascular disease [26, 27]. IR may exacerbate hypertension through increased sympathetic nervous system activity and enhanced renal sodium reabsorption, thereby increasing cardiac workload [28]. Hyperinsulinemia has also been associated with endothelial dysfunction and sympathetic activation [29, 30]. Given that the TyG index correlates with traditional cardiovascular risk factors such as obesity and hypertension [31, 32], a higher TyG index may indicate a more severe metabolic disturbance and consequently greater cardiovascular and overall mortality risk [33].

Existing evidence has demonstrated that an elevated TyG index was associated with increased risks of cardiovascular disease and diabetes [34, 35]. In individuals with cardiovascular disease and prediabetes or diabetes, the relationship between the TyG index and mortality risk has been reported to follow a U‐shaped pattern [35]. Studies in patients with coronary artery disease and hypertension have also reported associations between a higher TyG index and adverse outcomes [36, 37], supporting its potential prognostic relevance in cardiovascular disease. However, although the TyG index has been widely investigated across diverse populations, evidence specifically focusing on individuals with coexisting diabetes and hypertension remains limited. In our study, we observed a nonlinear association between TyG index and mortality risk in this high‐risk population, with risk increasing beyond internally identified inflection points derived from data‐driven modeling. It should be noted that these inflection points were estimated within the current cohort and should not be interpreted as clinically established cutoff values. External validation in independent cohorts is required before these values can be considered for clinical application. Previous studies in the general population have reported increased mortality risk at higher TyG levels. Notably, Liu et al. observed a nonlinear association between TyG index and mortality, with cardiovascular risk increasing when TyG exceeded approximately 9.52 in a general population cohort [38]. Similarly, Zhao et al. reported elevated cardiovascular mortality risk at TyG levels above 9 in middle‐aged and older adults with type 2 diabetes in the United States [39]. Although the threshold ranges appear numerically comparable to our internally identified inflection points, differences in study design and population characteristics preclude their interpretation as externally validated clinical cutoff values. Similar associations have also been observed in specific subgroups, such as middle‐aged and older adults in the United States [40]. Notably, Shen et al.’s study delved into the unique population of patients with diabetes and acute coronary syndrome, revealing that a higher TyG index was predictive of increased all‐cause mortality risk [41]. These findings further support the potential prognostic relevance of the TyG index in high‐risk populations. However, data specifically addressing nonlinear associations between TyG index and mortality in individuals with coexisting diabetes and hypertension remain limited. Our findings extend the existing evidence by examining this high‐risk population. Nonetheless, in the subgroup with the TyG index below the identified inflection points, our research failed to establish a link between TyG index and mortality risk, consistent with previous reports [35, 42]. This does not necessarily imply absence of association, as reverse causation cannot be excluded. In particular, lower TyG levels may reflect poor nutritional status, unintentional weight loss, sarcopenia, or underlying frailty, especially among older adults. Such conditions are independently associated with increased mortality risk and may confound the observed relationship between the TyG index and mortality. Therefore, the apparent lack of association at lower TyG levels should be interpreted cautiously, and future studies incorporating more detailed assessments of nutritional status and frailty are warranted. Differences in study design and population characteristics may also contribute to variability across studies [43].

We conducted stratified and interaction analyses to further explore the association between the TyG index and mortality risk. Among individuals younger than 65 years, a higher TyG index was associated with an increased risk of all‐cause mortality. Younger participants were more likely to have adverse cardiometabolic profiles, including higher obesity prevalence, elevated LDL‐C levels, greater alcohol consumption, and lower use of lipid‐lowering medications. The association remained after multivariable adjustment for these potential confounders. Although an interaction by age was observed (*p* for interaction = 0.01), this finding should be interpreted cautiously. Subgroup analyses may be limited by reduced statistical power, and CIs were relatively wide, particularly among younger participants. Furthermore, given that multiple subgroup analyses were conducted, the possibility of type I error cannot be excluded. Therefore, the age‐stratified findings should be considered exploratory rather than definitive. Given the observational design of this study, the findings reflect associations rather than causal relationships. From a clinical perspective, this finding may suggest potential clinical utility of the TyG index as a practical and readily available marker for early risk identification and stratification in younger individuals with coexisting diabetes and hypertension. However, these findings should not be interpreted as evidence of causality, and further studies are needed to determine whether incorporating TyG into risk assessment frameworks improves clinical outcomes.

### 4.1. Strengths and Limitations

This study has several strengths. First, although just a preliminary exploration, our study suggests that elevated TyG levels may be independently associated with increased risk of all‐cause and cardiovascular mortality among the high‐risk population with coexisting diabetes and hypertension. This finding helps bridge, to some extent, the gap in existing research regarding the association between the TyG and all‐cause/cardiovascular mortality risks among this specific patient population. Second, our study evaluated both linear and nonlinear associations between variables, enabling a more comprehensive understanding of the intricate link between the TyG index and mortality risks. Particularly, by identifying segmental effects and calculating precise identified inflection points, we provided more refined reference points for future research and clinical practice. Third, the effect modification analysis helped identify high‐risk subgroups precisely. Stratified analysis revealed a significant association between elevated TyG index and increased risk of all‐cause mortality among younger individuals.

Several limitations should also be acknowledged. First, owing to the observational study design, causal inferences cannot be established, and residual confounding may persist despite extensive multivariable adjustment. Second, the TyG index was assessed at baseline only, precluding evaluation of long‐term exposure or temporal changes in TyG levels. Third, fasting triglyceride and glucose measurements are subject to biological variability and potential analytical measurement error. Fourth, as this study was based on NHANES data representing the U.S. population, the generalizability of our findings to other populations or healthcare settings should be interpreted with caution. In addition, although the definitions of diabetes and hypertension incorporated objective laboratory measurements and standardized blood pressure assessments, some information, including physician diagnoses, medication use, and family history, relied on self‐reported data, which may introduce recall bias and potential misclassification. Despite adjustment for multiple confounders, residual or unmeasured confounding cannot be entirely excluded. However, all E‐values were greater than 2, indicating that an unmeasured confounder would need to be associated with both the TyG index and all‐cause or cardiovascular mortality with a risk ratio exceeding 2, independent of the adjusted covariates, in order to fully explain away the observed associations.

## 5. Conclusion

Among individuals with coexisting diabetes and hypertension, the TyG index showed a nonlinear relationship with all‐cause and cardiovascular mortality, with increased risks observed above internally identified inflection points at approximately 8.9 and 9.0, respectively. Elevated TyG levels were particularly associated with higher all‐cause mortality in patients younger than 65 years. Further studies are needed to determine whether incorporation of the TyG index improves risk prediction beyond established cardiovascular risk models.

### 5.1. Note

Preliminary findings from this study were previously presented in conference abstract/poster form and published in conference supplements [17, 18]. These earlier materials were limited to brief preliminary reports. In contrast, the current submission is a substantially expanded full‐length manuscript with a complete description of the study design, detailed methods, full statistical analyses, comprehensive results, and a substantially developed discussion and interpretation. These related materials are cited here for transparency and to clarify the differences between the prior conference publications and the present journal submission.

## Author Contributions

Conception and design of the study: all authors. Manuscript drafting: Yuanyuan Zhao and Dongjie Du. Statistical analysis: Yuanyuan Zhao and Dongjie Du. The acquisition of data: Dongjie Du. Critical revision of the manuscript for important intellectual content: all authors. Supervision: Zhi Liu.

## Funding

This study was supported by the National Natural Science Foundation of China (62172288).

## Disclosure

All authors read and approved the final manuscript.

## Ethics Statement

All NHANES protocols received approval from the ethical review committee of the National Center for Health Statistics.

## Conflicts of Interest

The authors declare no conflicts of interest.

## Supporting Information

Additional supporting information can be found online in the Supporting Information section.

## Supporting information


**Supporting Information** Supporting 1. Table S1. HRs (95% CIs) for mortality according to the TyG index using time‐dependent Cox regression models. Supporting 2. Table S2. Threshold effect analysis of the TyG index on all‐cause and cardiovascular mortality in diabetes patients with hypertension using time‐dependent Cox regression models. Supporting 3. Table S3. Missing data pattern of baseline characteristics. Supporting 4. Table S4. Baseline characteristics according to the TyG index tertiles after multiple imputation. Supporting 5. Table S5. HRs (95% CIs) for mortality according to the TyG index after multiple imputation. Supporting 6. Table S6. Threshold effect analysis of the TyG index on all‐cause and cardiovascular mortality in diabetic patients with hypertension after multiple imputations. Supporting 7. Table S7. HRs (95% CIs) for mortality according to the TyG index using multiple imputations for missing data and Fine‐Gray models for competing risks. Supporting 8. Table S8. HRs (95% CIs) for mortality according to the TyG index with the exclusion of deaths in the first year (*N* = 3237).

## Data Availability

The data are publicly available at https://www.cdc.gov/nchs/nhanes/index.html. To enhance transparency and reproducibility, the analytic code used in this study is available from the corresponding author upon reasonable request.
