# Supplementary material for: Nonlinear Relationship Between the Triglyceride–Glucose Index and All‐Cause and Cardiovascular Mortality in Diabetes Mellitus Patients With Hypertension: A National Cohort Study
Source: Int J Endocrinol. 2026 May 20;2026:4467241. doi: 10.1155/ije/4467241 (PMC13189463; doi:10.1155/ije/4467241)
Supplement: Supplementary file 1 — Supporting Information Supporting 1. Table S1. HRs (95% CIs) for mortality according to the TyG index using time‐dependent Cox regression models. Supporting 2. Table S2. Threshold effect analysis of the TyG index on all‐cause and cardiovascular mortality in diabetes patients with hypertension using time‐dependent Cox regression models. Supporting 3. Table S3. Missing data pattern of baseline characteristics. Supporting 4. Table S4. Baseline characteristics according to the TyG index tertiles after multiple imputation. Supporting 5. Table S5. HRs (95% CIs) for mortality according to the TyG index after multiple imputation. Supporting 6. Table S6. Threshold effect analysis of the TyG index on all‐cause and cardiovascular mortality in diabetic patients with hypertension after multiple imputations. Supporting 7. Table S7. HRs (95% CIs) for mortality according to the TyG index using multiple imputations for missing data and Fine‐Gray models for competing risks. Supporting 8. Table S8. HRs (95% CIs) for mortality according to the TyG index with the exclusion of deaths in the first year (N = 3237). [file IJE-2026-4467241-s001.docx]

| **Table S1. HRs (95% CIs) for mortality according to the TyG index using time-dependent cox regression models** | | | | | | | | | |
| --- | --- | --- | --- | --- | --- | --- | --- | --- | --- |
|  | **TyG (Continue)** | | **TyG (categories)** | | | | | | |
|  |  |  |  | **Q1**  **(6.80-8.85)** | **Q2**  **(8.85-9.40)** | | **Q3**  **(9.40-12.55)** | | ***p* for trend** |
| **All-cause mortality** | **HR (95%CI)** | ***p*** |  |  | **HR (95%CI)** | ***p*** | **HR (95%CI)** | ***p*** |  |
| Crude model | 0.96(0.88,1.05) | 0.34 |  | Ref. | 0.87(0.74,1.02) | 0.09 | 0.91(0.78,1.07) | 0.25 | 0.30 |
| Model 1 | 1.12(1.02,1.24) | 0.02 |  | Ref. | 0.89(0.76,1.05) | 0.18 | 1.14(0.97,1.34) | 0.12 | 0.09 |
| Model 2 | 1.20(1.05,1.38) | 0.008 |  | Ref. | 1.03(0.86,1.24) | 0.72 | 1.22(1.00,1.48) | 0.05 | 0.07 |
| **CVD mortality** |  |  |  |  |  |  |  |  |  |
| Crude model | 0.91(0.79,1.06) | 0.24 |  | Ref. | 0.80(0.61,1.05) | 0.11 | 0.81(0.62,1.06) | 0.13 | 0.14 |
| Model 1 | 1.13(0.95,1.33) | 0.16 |  | Ref. | 0.86(0.65,1.13) | 0.27 | 1.11(0.84,1.46) | 0.47 | 0.45 |
| Model 2 | 1.25(0.99,1.58) | 0.06 |  | Ref. | 1.01(0.74,1.37) | 0.96 | 1.23(0.88,1.71) | 0.28 | 0.23 |
| Crude model: Non-adjusted.  Model 1: Adjusted for age and race.  Model 2: Adjusted for age, race, gender, education, alcohol using, smoking status, BMI, LDL-C,  HDL-C, ALT, AST, TBiL, UA, familyCVD, familyDM, lipoprotein-lowering drugs, eGFR, CVD. | | | | | | | | | |

| **Table S2. Threshold effect analysis of TyG index on all-cause and cardiovascular mortality in diabetes patients with hypertension using time-dependent cox regression models** | | |
| --- | --- | --- |
|  | HR (95%CI) | *p* |
| **All-cause mortality** |  |  |
| Fitting by the standard Cox proportional risk model | 1.20(1.05, 1.38) | 0.008 |
| Fitting by two-piecewise Cox proportional risk model |  |  |
| Inflection point | 8.9 |  |
| TyG index< 8.9 | 0.87(0.63, 1.19) | 0.368 |
| TyG index≥ 8.9 | 1.40(1.16, 1.68) | <0.001 |
| *p* for Log-likelihood ratio | 0.028 |  |
| **Cardiovascular mortality** |  |  |
| Fitting by the standard Cox proportional risk model | 1.26(0.998, 1.591) | 0.052 |
| Fitting by two-piecewise Cox proportional risk model |  |  |
| Inflection point | 9.0 |  |
| TyG index< 9.0 | 0.64(0.39, 1.05) | 0.080 |
| TyG index≥ 9.0 | 1.75 (1.28,2.39) | <0.001 |
| *p* for Log-likelihood ratio | 0.005 |  |

| **Table S3. Missing data pattern of baseline characteristics.** | | |
| --- | --- | --- |
| **variables** | **Missing count** | **missing percent** |
| Age (year) | 0 | 0.00 |
| BMI, kg/m^2^ | 0 | 0.00 |
| SBP, mmHg | 111 | 3.37 |
| DBP, mmHg | 134 | 4.07 |
| TC, mmol/L | 0 | 0.00 |
| LDL-C, mmol/L | 177 | 5.37 |
| HDL, mmol/L | 0 | 0.00 |
| TG, mmol/L | 0 | 0.00 |
| eGFR, mL/min/1.73m^2^ | 15 | 0.46 |
| TyG | 0 | 0.00 |
| HbA1c, % | 9 | 0.27 |
| FPG, mg/dL | 0 | 0.00 |
| ALT, IU/L | 19 | 0.58 |
| AST, IU/L | 20 | 0.61 |
| TBiL, IU/L | 16 | 0.49 |
| UA, mmol/L | 14 | 0.42 |
| Race, n (%) | 0 | 0.00 |
| Gender (Male), n (%) | 0 | 0.00 |
| Education, n (%) | 9 | 0.27 |
| Alcohol, n (%) | 351 | 10.65 |
| Smoking status, n (%) | 6 | 0.18 |
| Hyperlipidemia, n (%) | 0 | 0.00 |
| Family CVD, n (%) | 0 | 0.00 |
| Family DM, n (%) | 0 | 0.00 |
| CVD, n (%) | 0 | 0.00 |
| Lipid-lowering drugs, n (%) | 3 | 0.09 |
| Anti-hypertension drug, n (%) | 3 | 0.09 |
| Glucose-lowering drug, n(%) | 3 | 0.09 |

| **Table S4.** Baseline characteristics according to the TyG index tertiles after multiple imputation | | | | | |
| --- | --- | --- | --- | --- | --- |
|  | **Tertiles of TyG index** | | | |  |
| **Variable** | **Total (n=3296)** | **Q1 (n=1102)** | **Q2 (n=1094)** | **Q3 (n=1100)** | ***p*** |
| Age, years | 63.28(12.77) | 64.97 (12.78) | 64.01(12.24) | 60.87 (12.94) | <0.001 |
| Male, n (%) | 1698 (51.5) | 573 (52.0) | 523 (47.8) | 602 (54.7) | 0.005 |
| Race, n (%) |  |  |  |  | <0.001 |
| Mexican American | 577 (17.5) | 126 (11.4) | 191 (17.5) | 260 (23.6) |  |
| Non-Hispanic Black | 845 (25.6) | 414 (37.6) | 236 (21.6) | 195 (17.7) |  |
| Non-Hispanic White | 1285 (39.0) | 372 (33.8) | 464 (42.4) | 449 (40.8) |  |
| Other Hispanic | 306 (9.3) | 85 (7.7) | 110 (10.1) | 111 (10.1) |  |
| Other race-  including multi-racial | 283 (8.6) | 105 (9.5) | 93 (8.5) | 85 (7.7) |  |
| Education, n (%) |  |  |  |  | 0.093 |
| <12 | 1174 (35.6) | 362 (32.8) | 392 (35.8) | 420 (38.2) |  |
| 12 | 983 (29.8) | 335 (30.4) | 322 (29.4) | 326 (29.6) |  |
| >12 | 1139 (34.6) | 405 (36.8) | 380 (34.7) | 354 (32.2) |  |
| Alcohol use, n (%) | 2706 (82.1) | 906 (82.2) | 898 (82.1) | 902 (82.0) | 0.991 |
| Smoking status, n (%) | 1662 (50.4) | 534 (48.5) | 534 (48.8) | 594 (54.0) | 0.014 |
| BMI, kg/m² | 32.22 (7.38) | 31.41 (7.96) | 32.50 (7.20) | 32.75 (6.89) | <0.001 |
| SBP, mmHg | 136.19 (20.25) | 135.76 (20.39) | 136.03 (20.13) | 136.79 (20.23) | 0.465 |
| DBP, mmHg | 70.25 (13.75) | 69.02 (13.69) | 70.25 (13.55) | 71.48 (13.91) | <0.001 |
| TC, mmol/L | 4.86 (1.19) | 4.46 (1.02) | 4.82 (1.05) | 5.29 (1.33) | <0.001 |
| LDL-C, mmol/L | 2.73 (1.00) | 2.55 (0.87) | 2.82 (0.95) | 2.83 (1.13) | <0.001 |
| HDL-C, mmol/L | 1.29 (0.40) | 1.48 (0.46) | 1.28 (0.31) | 1.10 (0.30) | <0.001 |
| Triglycerides, mmol/L | 1.88 (1.70) | 0.93 (0.30) | 1.57 (0.42) | 3.13 (2.41) | <0.001 |
| FPG, mg/dL | 151.95 (61.13) | 118.51 (25.16) | 140.82 (37.20) | 196.51 (77.20) | <0.001 |
| TyG, mean (SD) | 9.18 (0.75) | 8.42 (0.34) | 9.13 (0.16) | 10.00 (0.55) | <0.001 |
| HbA1c, % | 7.09 (1.70) | 6.44 (1.03) | 6.80 (1.26) | 8.03 (2.16) | <0.001 |
| eGFR, mL/min/1.73m² | 80.40 (25.36) | 78.76 (26.11) | 79.51 (24.60) | 82.94 (25.17) | <0.001 |
| ALT, IU/L | 26.54 (17.43) | 23.49 (14.16) | 26.18 (17.27) | 29.94 (19.80) | <0.001 |
| AST, IU/L | 26.26 (14.58) | 25.43 (13.85) | 25.86 (13.90) | 27.49 (15.84) | 0.002 |
| Total bilirubin, µmol/L | 11.76 (5.05) | 11.99 (5.29) | 11.58 (4.95) | 11.72 (4.91) | 0.149 |
| Uric acid, µmol/L | 354.26 (93.93) | 351.41 (92.60) | 355.04 (91.56) | 356.35 (97.53) | 0.442 |
| Hyperlipidemia, n (%) | 2901 (88.0) | 852 (77.3) | 974 (89.0) | 1075 (97.7) | <0.001 |
| Family CVD, n (%) | 495 (15.0) | 146 (13.2) | 176 (16.1) | 173 (15.7) | 0.128 |
| Family DM, n (%) | 2015 (61.1) | 657 (59.6) | 633 (57.9) | 725 (65.9) | <0.001 |
| CVD, n (%) | 905 (27.5) | 325 (29.5) | 276 (25.2) | 304 (27.6) | 0.081 |
| Lipoprotein-lowering  drugs, n (%) | 1649 (50.0) | 586 (53.2) | 539 (49.3) | 524 (47.6) | 0.028 |
| Anti-hypertension drug, n (%) | 2487 (75.5) | 855 (77.6) | 836 (76.4) | 796 (72.4) | 0.012 |
| Glucose-lowering drug (%) | 1918 (58.2) | 605 (55.0) | 623 (56.9) | 690 (62.8) | <0.001 |

TyG triglyceride-glucose index, BMI Body mass index, SBP Systolic blood pressure, DBP Diastolic blood pressure, HDL-C High-density lipoprotein cholesterol, LDL-C Low-density lipoprotein cholesterol, TC Total cholesterol, TG Triglyceride, UA Uric acid, FBG Fasting blood glucose, HbA1c Hemoglobin A1c, ALT Alanine aminotransferase, TBiL Total bilirubin, and AST Aspartate aminotransferase, eGFR Estimated glomerular filtration rate, CVD Cardiovascular disease.

| **Table S5. HRs (95% CIs) for mortality according to the TyG index** after multiple imputation**.** | | | | | | | | | |
| --- | --- | --- | --- | --- | --- | --- | --- | --- | --- |
|  | **TyG (Continue)** | | **TyG (categories)** | | | | | | |
|  |  |  |  | **Q1**  **(6.80-8.85)** | **Q2**  **(8.85-9.40)** | | **Q3**  **(9.40-12.55)** | | ***p* for trend** |
| **All-cause mortality** | **HR (95%CI)** | ***p*** |  |  | **HR (95%CI)** | ***p*** | **HR (95%CI)** | ***p*** |  |
| Crude model | 0.96(0.88,1.05) | 0.34 |  | Ref. | 0.87(0.74,1.02) | 0.09 | 0.91(0.78,1.07) | 0.25 | 0.30 |
| Model 1 | 1.13(1.02,1.24) | 0.01 |  | Ref. | 0.91(0.77,1.07) | 0.25 | 1.15(0.98,1.35) | 0.10 | 0.08 |
| Model 2 | 1.16(1.05,1.30) | 0.01 |  | Ref. | 0.93(0.78,1.10) | 0.41 | 1.17(0.98,1.41) | 0.08 | 0.05 |
| **CVD mortality** |  |  |  |  |  |  |  |  |  |
| Crude model | 0.91(0.79,1.06) | 0.24 |  | Ref. | 0.80(0.61,1.05) | 0.11 | 0.81(0.62,1.06) | 0.13 | 0.14 |
| Model 1 | 1.13(0.96,1.34) | 0.14 |  | Ref. | 0.87(0.66,1.15) | 0.32 | 1.12(0.85,1.47) | 0.44 | 0.42 |
| Model 2 | 1.20(0.99,1.45) | 0.05 |  | Ref. | 0.92(0.69,1.23) | 0.56 | 1.17(0.86,1.59) | 0.30 | 0.27 |
| Crude model: Non-adjusted.  Model 1: Adjusted for age and race.  Model 2: Adjusted for age, race, gender, education, alcohol using, smoking status, BMI, LDL-C,  HDL-C, ALT, AST, TBiL, UA, familyCVD, familyDM, lipoprotein-lowering drugs, eGFR, CVD. | | | | | | | | | |

| **Table S6. Threshold effect analysis of TyG index on all-cause and cardiovascular mortality in diabetes patients with hypertension** after multiple imputation | | |
| --- | --- | --- |
|  | HR (95%CI) | *p* |
| **All-cause mortality** |  |  |
| Fitting by the standard Cox proportional risk model | 1.16(1.05,1.30) | 0.01 |
| Fitting by two-piecewise Cox proportional risk model |  |  |
| Inflection point | 9.1 |  |
| TyG index< 9.1 | 0.83(0.66, 1.04) | 0.103 |
| TyG index≥ 9.1 | 1.37(1.19, 1.57) | <0.001 |
| *p* for Log-likelihood ratio | 0.001 |  |
| **Cardiovascular mortality** |  |  |
| Fitting by the standard Cox proportional risk model | 1.20(0.99,1.45) | 0.05 |
| Fitting by two-piecewise Cox proportional risk model |  |  |
| Inflection point | 9.0 |  |
| TyG index< 9.0 | 0.75(0.52, 1.10) | 0.144 |
| TyG index≥ 9.0 | 1.52 (1.20,1.93) | 0.001 |
| *p* for Log-likelihood ratio | 0.00 |  |

| **Table S7. HRs (95% CIs) for mortality according to the TyG index using multiple imputation**  **for missing data and Fine-Gray models for competing risks.** | | | | | | | | | |
| --- | --- | --- | --- | --- | --- | --- | --- | --- | --- |
|  | **TyG (Continue)** | | **TyG (categories)** | | | | | | |
|  |  |  |  | **Q1**  **(6.80-8.85)** | **Q2**  **(8.85-9.40)** | | **Q3**  **(9.40-12.55)** | | ***p* for trend** |
| **CVD mortality** |  |  |  |  |  |  |  |  |  |
| Crude model | 0.94(0.80,1.09) | 0.39 |  | Ref. | 0.82(0.63,1.08) | 0.16 | 0.84(0.65,1.10) | 0.20 | 0.22 |
| Model 1 | 1.16(0.98,1.38) | 0.09 |  | Ref. | 0.91(0.69,1.19) | 0.48 | 1.14(0.87,1.50) | 0.33 | 0.34 |
| Model 2 | 1.24(1.01,1.50) | 0.04 |  | Ref. | 0.96(0.72,1.28) | 0.22 | 1.21(0.89,1.64) | 0.22 | 0.21 |
| Crude model: Non-adjusted.  Model 1: Adjusted for age gender, and race.  Model 2: Adjusted for age, race, gender, education, alcohol using, smoking status, BMI, LDL-C,  HDL-C, ALT, AST, TBiL, UA, familyCVD, familyDM, lipoprotein-lowering drugs, eGFR, CVD. | | | | | | | | | |

| **Table S8. HRs (95% CIs) for mortality according to the TyG index  with exclusion of deaths in the first year (N=3,237).** | | | | | | | | | |
| --- | --- | --- | --- | --- | --- | --- | --- | --- | --- |
|  | **TyG (Continue)** | | **TyG (categories)** | | | | | | |
|  |  |  |  | **Q1**  **(6.80-8.85)** | **Q2**  **(8.85-9.40)** | | **Q3**  **(9.40-12.55)** | | ***p* for trend** |
| **All-cause mortality** | **HR (95%CI)** | ***p*** |  |  | **HR (95%CI)** | ***p*** | **HR (95%CI)** | ***p*** |  |
| Crude model | 0.98(0.90,1.07) | 0.640 |  | Ref. | 0.91(0.77,1.07) | 0.261 | 0.95(0.81,1.12) | 0.537 | 0.592 |
| Model 1 | 1.15(1.04,1.27) | 0.005 |  | Ref. | 0.94(0.80,1.12) | 0.512 | 1.19(1.00,1.41) | 0.044 | 0.033 |
| Model 2 | 1.21(1.05,1.39) | 0.009 |  | Ref. | 1.04(0.86,1.26) | 0.674 | 1.23(1.00,1.50) | 0.051 | 0.050 |
| **CVD mortality** |  |  |  |  |  |  |  |  |  |
| Crude model | 0.94(0.81,1.10) | 0.456 |  | Ref. | 0.87(0.66,1.16) | 0.340 | 0.87(0.66,1.15) | 0.324 | 0.344 |
| Model 1 | 1.18(1.00,1.40) | 0.056 |  | Ref. | 0.95(0.72,1.27) | 0.733 | 1.21(0.91,1.60) | 0.200 | 0.188 |
| Model 2 | 1.33(1.04,1.69) | 0.021 |  | Ref. | 1.07(0.78,1.46) | 0.693 | 1.31(0.93,1.86) | 0.120 | 0.131 |
| Crude model: Non-adjusted.  Model 1: Adjusted for age and race.  Model 2: Adjusted for age, race, gender, education, alcohol using, smoking status, BMI, LDL-C,  HDL-C, ALT, AST, TBiL, UA, familyCVD, familyDM, lipoprotein-lowering drugs, eGFR, CVD. | | | | | | | | | |
